# *Streptococcus pneumoniae* resists intracellular killing by olfactory ensheathing cells but not by microglia

**DOI:** 10.1038/srep36813

**Published:** 2016-11-09

**Authors:** Hugo Macedo-Ramos, Susana Ruiz-Mendoza, Rafael M. Mariante, Erick V. Guimarães, Lucas C. Quadros-de-Souza, Mauricio M. Paiva, Eliane de O. Ferreira, Tatiana C. A. Pinto, Lucia M. Teixeira, Silvana Allodi, Wagner Baetas-da-Cruz

**Affiliations:** 1 Laboratório Translacional em Fisiologia Molecular, Centro de Cirurgia Experimental, Faculdade de Medicina, Universidade Federal do Rio de Janeiro, Rio de Janeiro, RJ, Brazil; 2 Programa de Pós-Graduação em Ciências Biológicas (Fisiologia), Instituto de Biofísica Carlos Chagas Filho, Universidade Federal do Rio de Janeiro, Rio de Janeiro, RJ, Brazil; 3 Laboratório de Neurobiologia Comparativa e do Desenvolvimento, Instituto de Biofísica Carlos Chagas Filho, Universidade Federal do Rio de Janeiro, Rio de Janeiro, RJ, Brazil; 4 Laboratório de Neurogênese, Instituto de Biofísica Carlos Chagas Filho, Universidade Federal do Rio de Janeiro, Rio de Janeiro, RJ, Brazil; 5 Laboratório de Biologia Estrutural, Instituto Oswaldo Cruz, Fiocruz, Rio de Janeiro, Brazil; 6 Instituto Nacional de Tecnologia, Rio de Janeiro, RJ, Brazil; 7 Universidade Federal do Rio de Janeiro - Polo Xerém, Duque de Caxias, Rio de Janeiro, RJ, Brazil; 8 Instituto de Microbiologia Paulo de Góes, Universidade Federal do Rio de Janeiro, Rio de Janeiro, RJ, Brazil

## Abstract

Olfactory ensheathing cells (OECs) are a type of specialized glial cell currently considered as having a double function in the nervous system: one regenerative, and another immune. *Streptococcus pneumoniae* is a major agent of severe infections in humans, including meningitis. It is commonly found in the nasopharynx of asymptomatic carriers, and, under certain still unknown conditions, can invade the brain. We evaluated whether pneumococcal cells recovered from lysed OECs and microglia are able to survive by manipulating the host cell activation. An intracellular-survival assay of *S. pneumoniae* in OECs showed a significant number of bacterial CFU recovered after 3 h of infection. In contrast, microglia assays resulted in a reduced number of CFU. Electron-microscopy analysis revealed a large number of pneumococci with apparently intact morphology. However, microglia cells showed endocytic vesicles containing only bacterial cell debris. Infection of OEC cultures resulted in continuous NF-κB activation. The IFN-γ-induced increase of iNOS expression was reversed in infected OECs. OECs are susceptible to *S. pneumoniae* infection, which can suppress their cytotoxic mechanisms in order to survive. We suggest that, in contrast to microglia, OECs might serve as safe targets for pneumococci, providing a more stable environment for evasion of the immune system.

Olfactory ensheathing cells (OECs) are a type of specialized glial cell that accompany and ensheath the primary olfactory axons through the olfactory pathway, from the olfactory epithelium to the olfactory tract. OECs are essential for olfactory axonal outgrowth and guidance within the developing and adult olfactory system^1^,^2^. This property of OECs makes them an outstanding candidate for cellular therapy to stimulate central nervous system (CNS) repair after injury^3^. However, to create a favourable microenvironment for neurogenesis, the OECs must interact with the lesion site in order to avoid triggering more aggressive responses than those caused by the initial damage. Previous studies have focused on improving the understanding of the immunomodulatory mechanisms of OECs during neurological disorders, including those caused by pathogens^4^,^5^,^6^,^7^.

*Streptococcus pneumoniae* is a major bacterial agent of severe infections in humans, including meningitis. This microorganism is commonly found in the nasopharynx of asymptomatic carriers, and, under certain still largely unknown conditions, can become pathogenic and invade the CNS^8^,^9^. The mechanism by which some strains of *S. pneumoniae* gain access to the brain without being able to survive in the bloodstream remains unknown. Some evidence points to a non-hematogenous invasion of the brain by *S. pneumoniae,* through transport along the olfactory bulb (OB)^10^. Recent data from our group confirmed these findings, by detecting *S. pneumoniae* DNA in the OB of bacteria-challenged mice^11^. Although several lines of evidence indicate that *S. pneumoniae* reaches the OB, based on the use of molecular techniques for the detection of bacterial DNA and specific pneumococcal antigens, no data are available to support the idea that the bacteria can survive in the OB cells and therefore be able to spread the infection through the CNS. In the present study, we evaluated whether pneumococci recovered from lysed OECs and from microglia cells are able to survive by manipulating the host cell to favor their continuity in a less-hostile environment.

## Results

### *In vitro* infection of *S. pneumoniae* in OECs or microglia cultures

**Figure 1:**
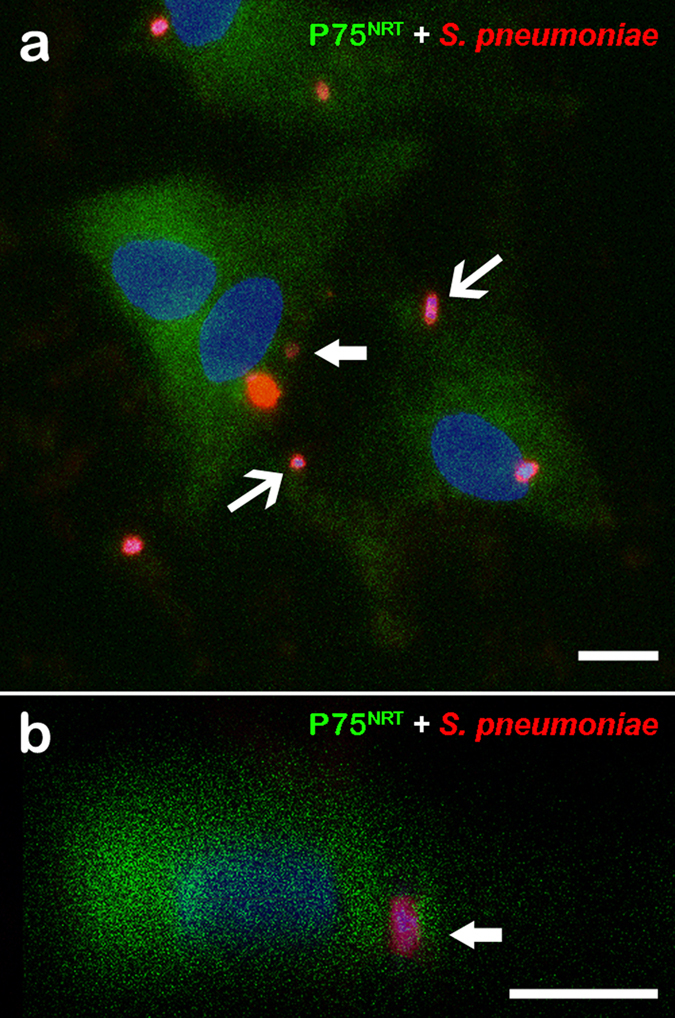
Confocal microscopy images showing expression of the phenotypic marker p75^NRT^ in olfactory ensheathing cells (OECs) infected by *Streptococcus pneumoniae*. (**a**) Optical section showing OEC cultures infected by *S. pneumoniae* for 3 h and immunolabeled for p75^NRT^ and Alexa 488-labeled secondary antibody. The nuclei of OECs and/or bacterial DNA (blue dots) were stained with DAPI. The DAPI counterstaining shows the bacterial DNA surrounded by intense labeling of the pneumococcal capsule by the anti-pneumococcal antiserum and Cy3-tagged secondary antibody (arrows in **a**,**b**). (**b**) Orthogonal plane image cut at the maximum nucleus diameter of a shows details of internalized *S. pneumoniae* (thick arrows). The data are representative of three separate experiments, each conducted in triplicate. Scale bar = 12 μm (**a**); 8 μm (**b**).

The occurrence of *S. pneumoniae* infection was analyzed after interaction with OECs for 3 h. *S. pneumoniae* was detected by using pneumococcal anti-serum. The results revealed a variable number of internalized bacterial cells throughout the cytoplasm of OECs, which were identified by the phenotypic marker p75^NRT^ (Fig. 1a,b).

**Figure 2:**
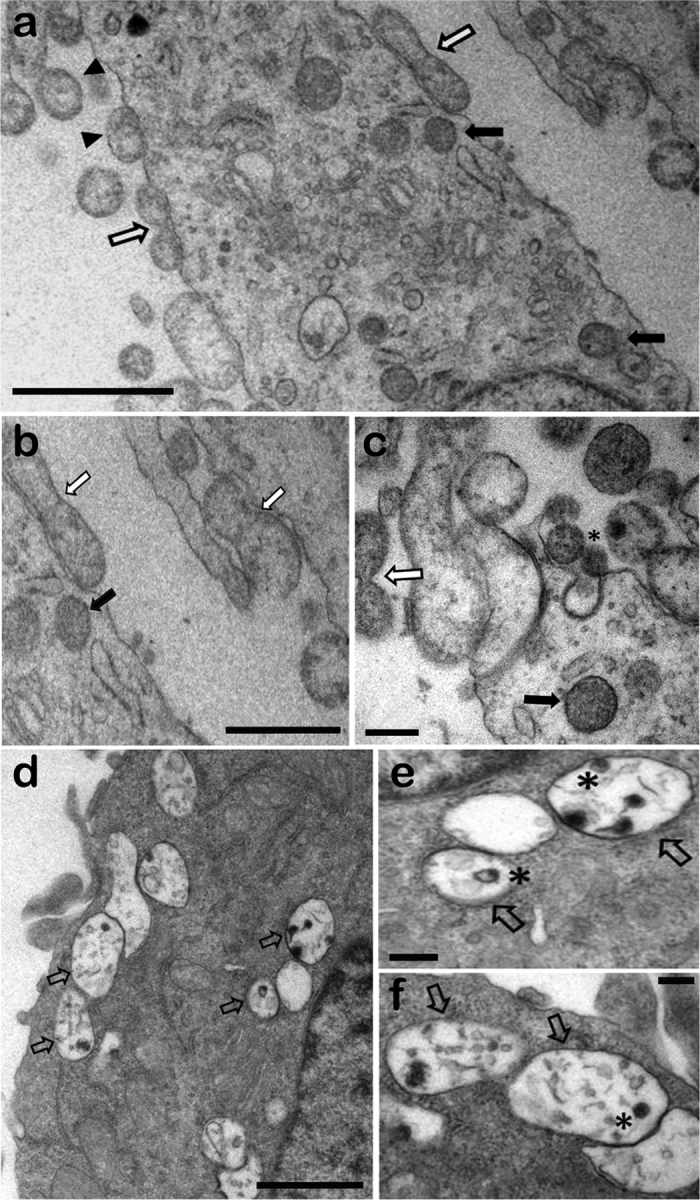
Transmission electron microphotographs of olfactory ensheathing cells (OECs) (a–c) and microglia (d–f) infected with *Streptococcus pneumoniae*. (**a**) An overview of different invasion stages, showing adherent pneumococcal cells associated with electron-dense material (arrowheads) and diplococcus-shaped pairs (white arrows), as well as intracellular pneumococci in vacuoles (black arrows). (**b**) Subsequent engulfment by cellular protrusions, with the formation of a membrane-bound vacuole. (**c**) Asterisk indicates a possible clathrin-coated pit-like structures and electron-dense material associated with invading bacteria. (**d**–**f**) Intracellular pneumococci in vacuoles showing clear signs of degradation. Higher-magnification views of digested bacteria in cytosolic vacuoles (asterisk in **e**,**f**). Scale bar = 1 μm (**a**); 0.7 μm (**b**); 0.2 μm (**c**); 1 μm (**d**); 0.2 μm (**e**); 0.2 μm (**f**).

Ultrastructural analysis of infected OECs or microglia cells by transmission electron microscopy revealed the presence of a large number of *S. pneumoniae* attached to the plasma membrane or internalized in endocytic vesicles in different regions of the OEC cytoplasm (Fig. 2a–c). The bacteria internalized in endocytic compartments of OECs showed apparently intact morphology (Fig. 2a,c), in striking contrast to that observed in N13 cells under the same conditions of infection (Fig. 2d–f). After 3 h of interaction with *S. pneumoniae,* N13 cells contained only bacterial cell debris within large vacuoles, compatible with intracellular digestion process (Fig. 2e,f).

### *S. pneumoniae* entry and survival in OECs but not in microglia

**Figure 3:**
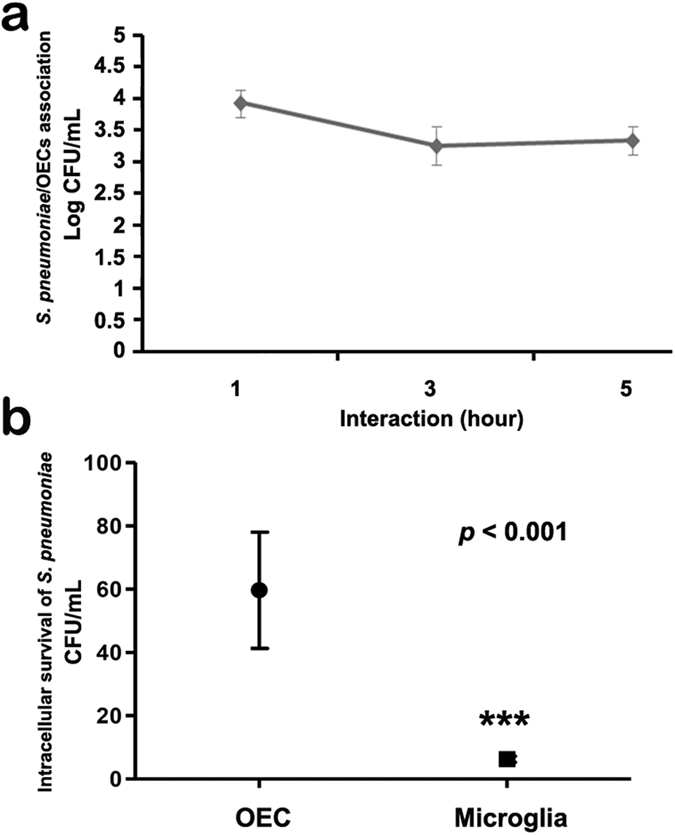
Kinetics of association (adhesion or internalization) with and survival of *Streptococcus pneumoniae* in OECs. (**a**) The percentage of OECs containing adhered or internalized *S. pneumoniae* was quantified at different times up to 5 h. The graph shows the number of OECs containing adhered and/or internalized *S. pneumoniae*, which did not vary over the time intervals. (**b**) Intracellular survival assay with the exclusion of *S. pneumoniae* attached to the plasma membrane after treatment with antibiotic, and bacteria recovery after 3 h of infection followed by lysis of the host cell. The graph shows a significant number of CFU from infected OECs compared to the N13 cells that reduced the number of CFU. For statistical analysis, we used One-way ANOVA and Tukey’s Multiple Comparison Test.

We evaluated the kinetics of association (adhesion or internalization) of *S. pneumoniae* with OECs or N13 cells, washing the infected cultures and then lysing them in buffers for different interaction times of 1, 3 and 5 h. The counts of the colonies formed after plating the OECs lysates showed no significant differences between these interaction times, revealing that the number of OECs containing adhered and/or internalized *S. pneumoniae* did not vary over short intervals of interaction (Fig. 3a).The intracellular survival assay with the exclusion of *S. pneumoniae* attached to the plasma membrane of OECs through treatment with an antibiotic showed a significant number of CFU from bacteria recovered after 3 h of infection, followed by lysis of the host cell (Fig. 3b). On the other hand, the same test performed with N13 cells resulted in a reduced number of CFU from bacteria recovered after 3 h of infection (Fig. 3b).

### *S. pneumoniae* induces continuous NF-κB p65 activation in OECs

**Figure 4:**
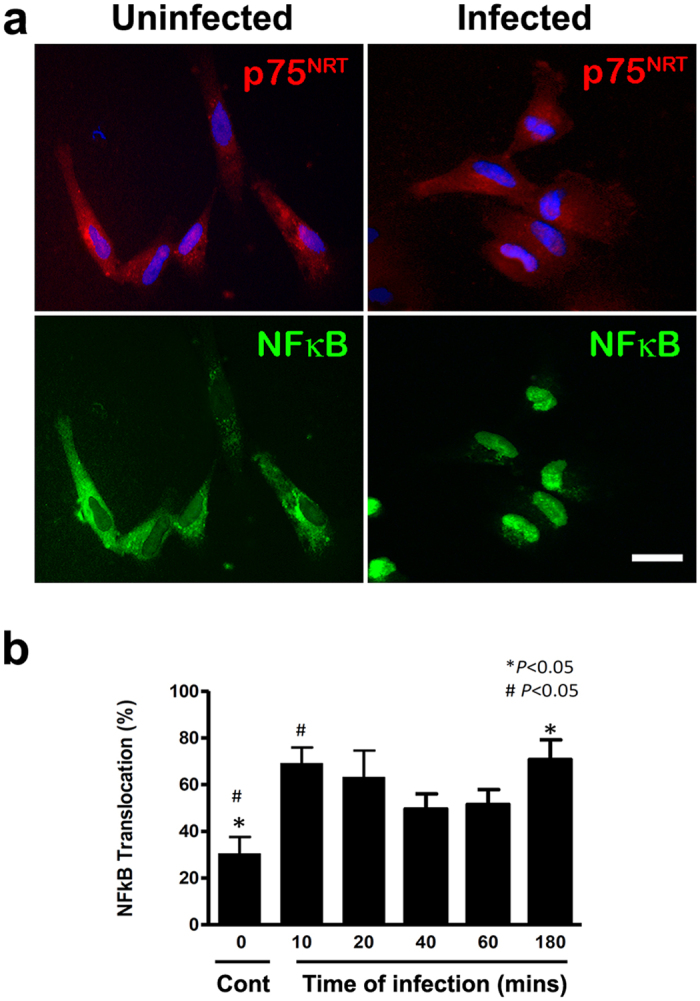
Cellular distribution of NF-κB p65 in OEC cultures uninfected and infected by *Streptococcus pneumoniae*. (**a**) Lower left panel: shows absence of nuclear translocation of NF-κB-p65 in OECs maintained in their normal culture medium alone. Lower right panel: infection of the OEC cultures with *S. pneumoniae* for up to 3 h resulted in massive nuclear translocation of NF-κB. (**b**) Infection of the OEC cultures with *S. pneumoniae* resulted in continuous nuclear translocation of NF-κB. The nuclei of the cells were stained with DAPI. The data are representative of three separate experiments, each conducted in triplicate. For statistical analysis, we used One-way ANOVA and Tukey’s Multiple Comparison Test. Scale bar = 20 μm.

Infection of the OEC cultures with *S. pneumoniae* for up to 3 h resulted in continuous nuclear translocation of NF-κB. After 10 min of infection, the majority of the OECs showed a massive nuclear translocation of NF-κB. In contrast, intense NF-κB labeling was observed in the cytoplasm from control uninfected OEC cultures maintained under the same conditions (Fig. 4a,b).

### *In vitro* regulation of iNOS expression in OECs

**Figure 5:**
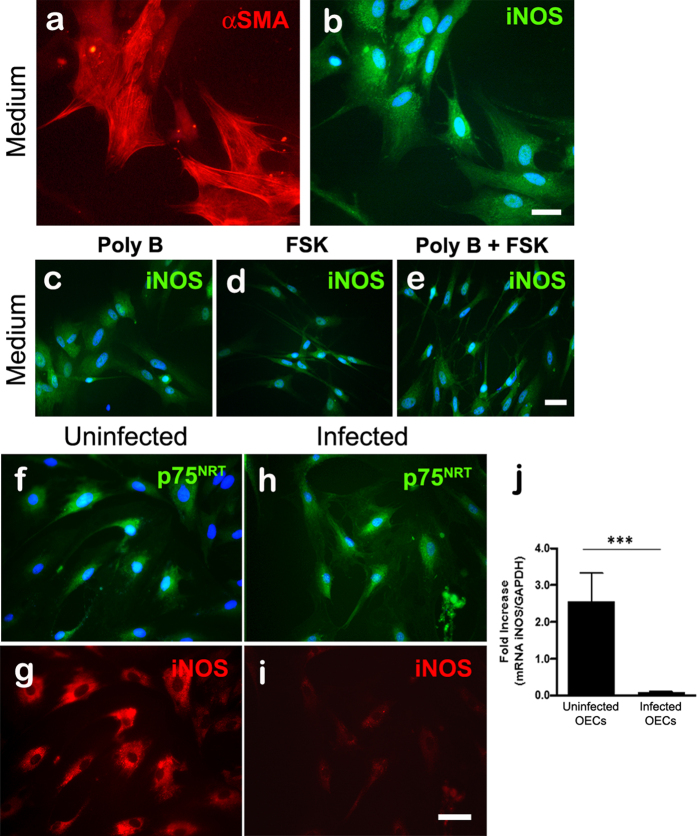
*In vitro* regulation of iNOS expression during infection of OECs by *Streptococcus pneumoniae.* (**a**,**b**) OECs phenotypically identified by αSMA expressed iNOS constitutively *in vitro*. (**c**) Polymyxin B (poly B) was used as an inhibitor of LPS contamination, and the treatment of OEC cultures did not induce significant differences in the expression of iNOS. Forskolin (FSK) was added to the defined media as culture requirements for OEC cultures, and is also described as a costimulatory agent of the LPS-induced iNOS expression. (**d**,**e**) FSK alone or in combination with poly B had little or no effect on iNOS expression. *S. pneumoniae* infection of OECs, phenotypically identified by their expression of P75^NRT^, reduced the expression of iNOS (**h**,**i**) compared to uninfected cultures (**f**,**g**). The nuclei of the cells were stained with DAPI. (**j**) Quantitative RT-PCR analysis of iNOS mRNA in uninfected and infected OEC cultures with *S. pneumoniae*. The graph shows a significant reduction of mRNA levels of iNOS in infected cultures compared to uninfected control OEC cultures. The s.d. values are shown, n = 4; stars indicate significance in two-tailed Student’s *t*-test; ***P < 0.001. All experiments were run in triplicate or quadruplicate, and each experiment was repeated two or three times. Scale bar = 25 μm (**a**,**b**); 45 μm (**c**–**e**); 30 μm (**f**–**i**).

**Figure 6:**
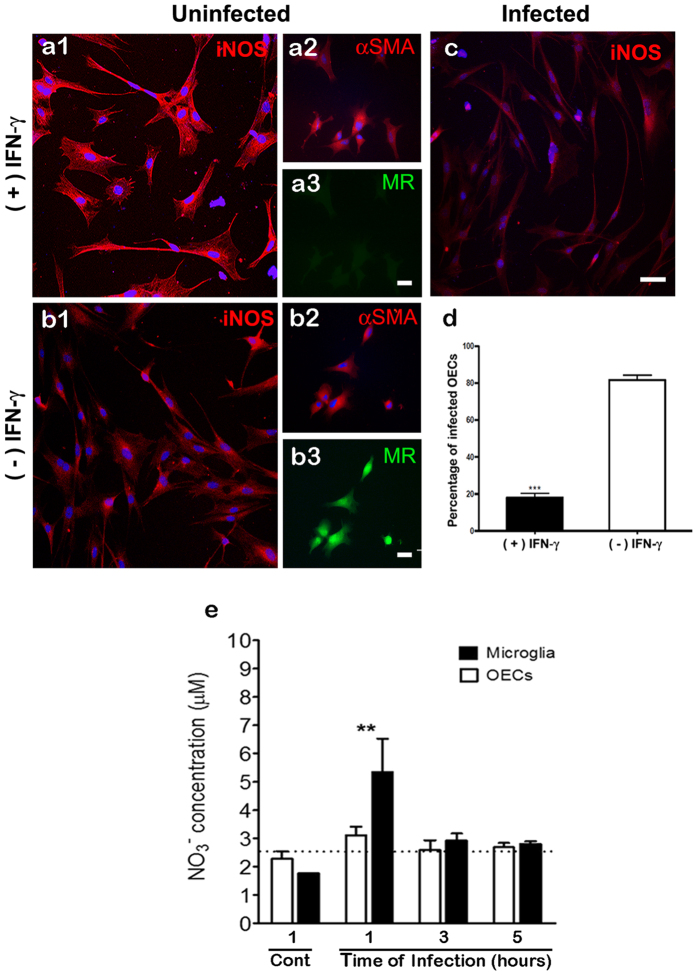
*Streptococcus pneumoniae* modulates iNOS expression by OECs IFN-γ-treated. Treatment of the cultures with rIFN-γ (100 U/mL) resulted in a substantial increase in iNOS expression by OECs after 18 h of stimulation (**a1**) compared to untreated OEC cultures (**b1**). On the other hand, this increase in iNOS expression IFN-γ-induced was reversed in cultures infected by *S. pneumoniae* for an additional period of 3 h (**c**). Treatment of cultures with IFN-γ for 18 h prior to beginning the protocol of infection by *S. pneumoniae* significantly reduced the percentage of infected OECs, compared to control cultures maintained during the same period in medium alone (**d**). Treatment of OECs with IFN-γ for 18 h significantly reduced the expression of Mannose Receptor (MR) (**a3**) compared to the untreated control cultures (**b3**). (**e**) *S. pneumoniae* affects the nitrite levels in OECs or N13 cells. Neither uninfected nor infected OEC cultures increased their production of NO (black bar), which was below the detection limit in all time intervals. In contrast, N13 microglia showed a significant increase in NO levels after 1 h of infection, but NO subsequently declined to below the detection limit at 3 and 5 h of infection (white bar). The nuclei of the cells were stained with DAPI. The data are representative of three separate experiments, each conducted in triplicate. Scale bar = 50 μm (**a1**,**b1**,**c**); 30 μm (**a3**,**b3**).

In our culture system, the OECs phenotypically identified by the expression of αSMA were capable of expressing iNOS even in the absence of any stimulus (Fig. 5a,b). We observed no significant differences in the expression of iNOS after treatment of OECs with poly B (Fig. 5c). Incubation of OECs with FSK appears to have had little or no effect (Fig. 5d). Furthermore, OEC cultures incubated with FSK showed a marked change in morphology, displaying an elongated fusiform shape (Fig. 5d). This morphological pattern was not changed by polyB (Fig. 5e). *S. pneumoniae* infection of OECs, phenotypically identified by the expression of P75^NRT^, was able to reduce the expression of iNOS (Fig. 5h,i) compared to uninfected cultures (Fig. 5f,g). This negative regulation of expression of the iNOS by *S. pneumoniae* was confirmed by quantitative RT-PCR analysis; iNOS expression was significantly higher in uninfected control OEC cultures than in infected cultures in the same conditions for 3 h (Fig. 6j). Incubation of the microglia cultures with poly B or FSK did not result in any changes similar to those observed in the OEC cultures (data not shown).

### *S. pneumoniae* modulates iNOS expression by OEC IFN-γ-treatment

Because iNOS is involved in the responses to host cell infection by *S. pneumoniae*, we tested whether iNOS could be modulated during infection of OECs or N13 cells. Treatment of the cultures with rIFN-γ (100 U/mL) resulted in a substantial increase in iNOS expression by OECs after 18 h of stimulation (Fig. 6a1) compared with untreated OEC cultures (Fig. 6b1). On the other hand, the increase in IFN-γ-induced iNOS expression was reversed in cultures infected by *S. pneumoniae* for an additional period of 3 h (Fig. 6c).

Treatment of cultures with IFN-γ for 18 h prior to infection by *S. pneumoniae* significantly reduced the percentage of infected OECs, compared to control cultures maintained for the same period in medium alone (Fig. 6d). These data suggest a reduction in the endocytic activity of the OECs via downregulation of Mannose Receptor (MR) expression, considering that proinflammatory reagents, such as interferon-gamma (IFN-γ), suppress this expression^12^. To test this hypothesis, we treated OEC cultures with IFN-γ and then added the MR antibody. Treatment of OECs with IFN-γ for 18 h significantly reduced the expression of MR (Fig. 6a3) compared to the untreated control cultures (Fig. 6b3). Since iNOS is involved in the production of microbicidal products such as NO, we investigated whether infection by *S. pneumoniae* affects the levels of NO in OECs or N13 cells. Neither uninfected nor infected OEC cultures increased the production of NO, which remained below the detection limit in all the time intervals. In contrast, N13 microglia showed a significant increase in NO levels after 1 h of infection, but NO levels then declined to below the detection limit after 3 and 5 h of infection (Fig. 6e).

## Discussion

Previous studies have shown that the OECs increase the *in vitro* content of iNOS after challenge by bacteria such as *Escherichia coli* and *Staphylococcus aureus*^5^,^6^. In opposition to other reports, our data revealed that OECs infected with *S. pneumoniae* for a period of 3 h showed an apparent reduction of iNOS levels compared to uninfected OECs. In spite of this, our results suggest that *S. pneumoniae* may negatively regulate iNOS, allowing the establishment of infection. As far as we are aware, OECs do not appear to have the same microbicidal activity of professional phagocytes, failing to destroy the internalized bacteria. The down-regulation of iNOS expression in OECs induced through the *S. pneumoniae* infection can be attributed to a variety of factors, including: i) the ability of these bacteria to cause protein degradation, in this case, iNOS; ii) the ability of *S. pneumoniae* to secrete endonucleases to cleave the mRNA sequences involved in transcriptional regulation of iNOS^13^.

This biphasic response of OECs to different groups of bacteria can be attributed to the characteristics of each of them, and in our case it is not surprising that highly pathogenic strains of *S. pneumoniae* such as EF3030 are capable of suppressing the cellular activation mechanism. The same situation may not occur in professional phagocytes, such as microglia, where pathogenic bacteria would be rapidly killed and efficiently degraded after they invade, preventing the establishment of infection.

The OECs obtained by our culture method expressed iNOS constitutively. This finding was unexpected since this protein requires stimulation by endogenous or exogenous agents for its expression in non-professional phagocytes. Initially, we suspected contamination by LPS, but this possibility was discarded as incubation of the cultures with poly B did not alter the results. In another experiment, we found no substantial differences between this “constitutive” and induced (stimulation with 100 U/mL IFN-γ) expression of iNOS by OECs. However, additional studies are necessary to better understand this mechanism involved in the activation of iNOS in OECs.

NF-κB is an important transcription factor involved in the synthesis of proinflammatory molecules such as iNOS. Therefore, NF-κB translocation is essential to produce iNOS, and consequently to release NO from the cell. Inorganic anion nitrite (NO_2_^−^) is an end product of endogenous NO metabolism, and its presence has a crucial role in NF-κB inhibition^14^. As long as NO is produced, the concentration increases, preventing NF-κB translocation. We evaluated the translocation of NF-κB in order to test whether this might be one of the mechanisms by which the pneumococci can downregulate the expression of iNOS, considering that it was previously reported that NF-κB remains translocated in the absence of NO. In fact, Harris *et al*. showed that NF-κB remains translocated in the presence of inhibitors of NO production, suggesting the existence of a negative feedback loop between NF-κB and NO^6^. Our studies with NF-κB activation during the kinetics of the association of *S. pneumoniae* with OECs showed that it remains translocated in the nucleus for 10 to 180 min of interaction. These results are consistent with the reduced expression of iNOS and low nitrite levels found in infected OEC cultures. Collectively, these data suggest a possible mechanism triggered by *S. pneumoniae* that is able to suppress the pathway for expression of iNOS and therefore the production of NO. Furthermore, recent data from our group suggest the existence of a soluble factor that is generated after interaction of *S. pneumoniae* with OECs, which can simultaneously be able to regulate NF-κB activation and the viability of microglia^11^. However, continued studies are necessary to better understand the influences of *S. pneumoniae* on the biosynthetic pathway for NO production, and its impact on continuous NF-κB p65 activation in OECs.

It is largely known that proinflammatory cytokines such as IFN-γ negatively regulate the expression and activity of MR^12^,^15^; and we found a similar result in OECs, with a drastic reduction of MR expression after treatment with IFN-γ. Our findings showed that the percentage of OECs infected by *S. pneumoniae* decreased substantially after the cultures were treated for 18 h with IFN-γ, supporting our previous data indicating a possible involvement of MR in the internalization of *S. pneumoniae*. Nevertheless, we cannot rule out the possibility that other factors are involved in the reduction of infection, such as the activation of iNOS also induced by INF-γ.

The intracellular viability assay revealed a large number of CFU of *S. pneumoniae* internalized by the OECs, at different interaction times (1, 3 and 5 h). This contrasted with the results obtained for microglia cells, in which only a small number of CFU were observed, demonstrating that OECs can provide a much more permissive environment for intracellular survival of *S. pneumoniae*. In these assays we found that the viability of *S. pneumoniae* was not compromised, and also there were no significant differences among the times evaluated, which reinforces our hypothesis that OECs function as host cells for this pathogen. Supporting this hypothesis, our results showed that pneumococcal infection drives microglia into a potentially lethal function, increasing NO levels, which in turn can be suppressed by reduction of the infection load. These data contrast markedly with our observations on OECs, which remained unresponsive and did not increase their production of NO during the course of the infection.

Ultrastructural analysis after interactions for 3 h showed adherent pneumococci on the cellular surface and also in plasma membrane expansions, suggestive of recent engulfment by OECs due to self-induced bacterial invasion. Three hours after the interaction, a few vacuoles in the cytoplasm of the microglia were observed, containing still-undigested bacteria. Thus, the gradual decrease in bacterial load could be related to a low ability of microglia to harbor *S. pneumoniae* infection.

Recent studies support the hypothesis that OECs could play a role in the innate immune response, assuming a role in the front-line defense of the olfactory system against bacterial infections, due to their ability to detect and confine pathogens^4^,^5^,^16^. Olfactory ensheathing cells (OECs) are a type of specialized glial cell, currently considered as having a double function in the nervous system: one regenerative, essential for axonal outgrowth and guidance; and another immune, as phagocytes involved in the engulfment of pathogens and cell debris^16^,^17^. Although they are not considered professional phagocytes, OECs seem to play an important role in tissue clearance, considering that they have a range of receptors for pathogen-associated molecular patterns (PAMPs) and damage-associated molecular patterns (DAMPs)^4^,^5^,^7^.

Our data provide some clues about a possible OEC function as a mediator of *S. pneumoniae* invasion of the CNS. Pneumococcal meningitis usually develops through various stages of interaction between the bacteria and the host cell, including the initial steps, from the survival of pneumococci and the subsequent invasion of the CNS^18^.

**Figure 7:**
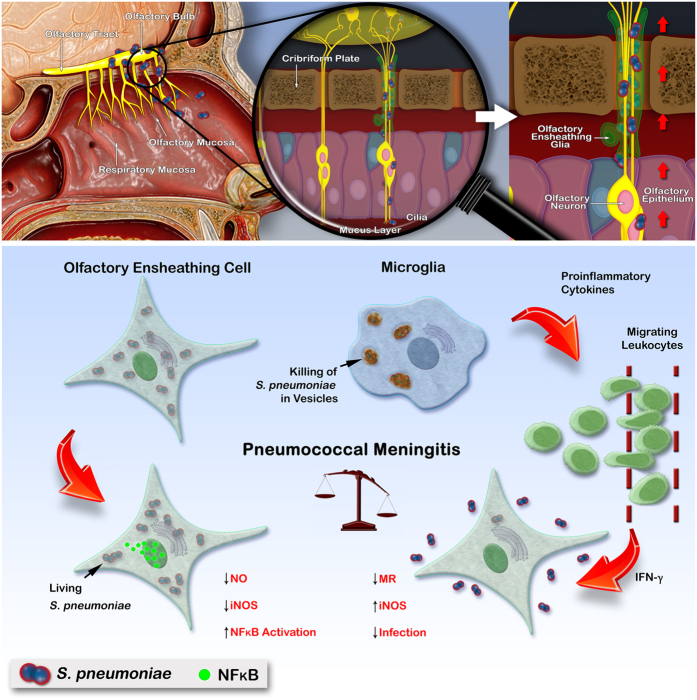
Schematic representation of the olfactory system as a potential entry portal for non-hematogenous invasion of *Streptococcus pneumoniae* into the brain, via an olfactory ensheathing glia route. The nasal cavity, olfactory mucosa and OB are shown (upper left panel). The axon bundles merge to form a large nerve extending from the olfactory epithelium through the cribriform plate into the OB immediately adjacent (upper middle panel). The diagram shows a hypothetical scheme for microglia activation, by which ingested *S. pneumoniae* are killed rapidly (lower panel). The OECs (green) surrounding these axon bundles become infected, and from there the pneumococci (blue spheres in lower panel) could spread throughout the OB. Differently from microglia, OECs are susceptible to infection by *S. pneumoniae*, which is capable of suppressing their cytotoxic mechanisms in order to survive. A putative paracrine mediator released by infected microglia may directly affect leukocyte migration, which in turn modulates the activation of the OECs (lower panel).

Taken together, the results of the present study showed for the first time that *S. pneumoniae* resists intracellular killing and prevents OECs activation. Increased intracellular survival of *S. pneumoniae* may be a passive strategy to evade the host’s microbicidal mechanisms, avoiding attack by immune cells such as microglia, by hiding the bacterium inside the OECs (Fig. 7). Continued studies are necessary to further elucidate the immune defense mechanisms regulating the function of OECs and their interaction with *S. pneumoniae*, as they represent a potential line of defense against infectious pathogens in the OB. On the other hand, the presence of a glial cell type near axons projecting from the olfactory epithelium to the OB, susceptible to the counter-regulation of its immunological function by pathogens, could create a route that potentially renders the CNS more vulnerable to infection by *S. pneumoniae* strains that may fail to survive in the bloodstream.

## Methods

### Primary OECs cultures

Primary rat OEC cultures were obtained by the procedure described by Nash *et al*.^19^, as modified by our group^7^,^20^. Eight-week-old male Wistar rats were obtained from the Center for Experimental Surgery Laboratory at the Federal University of Rio de Janeiro. Animal care, all experiments, and euthanasia were approved by the Ethics Committee for Laboratory Animal Use in Research of the Federal University of Rio de Janeiro (CEUA - UFRJ), Permit Number: 01200.001568/2013–87 in accordance with the International Guiding Principles for Biomedical Research Involving Animals, recommended by the World Health Organization (Committee, 2011)^21^. The cells were plated in the desired density into the appropriate laminin-coated flasks (Sigma Chemical, St. Louis, MO, USA), plates or wells in DMEM/F12 medium (Invitrogen, Carlsbad, CA, USA) supplemented with 10% heat-inactivated fetal calf serum (FCS; Cultilab, Campinas, SP, Brazil), 2 μM forskolin (FSK; Calbiochem, La Jolla, CA, USA), and 20 μg/mL bovine pituitary extract (BPE – Biomedical Technologies, Massachusetts, MA, USA), 1 mM glutamine (Biofluids Inc., Rockville, Maryland, USA), 100 U/mL penicillin, and 50 μg/mL streptomycin (Invitrogen, Carlsbad, CA, USA). The cells were kept at 37 °C in 5% CO_2_, and the medium was changed regularly.

### Microglia cell line culture

The N13 microglia cell line^22^ was kindly provided by Dr. Claudia Verderio (Department of Medical Pharmacology, CNR Institute of Neuroscience, University of Milan, Milan, Italy). Cells were cultured in RPMI 1640 medium (Thermo Fisher Scientific, Waltham, MA, USA) supplemented with 2 mM glutamine, 10% fetal calf serum, 100 U/mL penicillin and 50 μg/mL streptomycin. Flasks were maintained at 37 °C in a humidified atmosphere containing 5% CO_2_.

### Pneumococcal strain

The encapsulated strain of *S. pneumoniae* (EF3030, serotype 19F) used in the present study was kindly donated by Dr. David E. Briles (University of Alabama, Birmingham, AL, USA). The EF3030 strain was selected for performing interaction assays with OECs based on the fact that it colonizes the upper respiratory tract in the absence of spreading through the bloodstream^23^ and it belongs to a serotype frequently associated with resistance to penicillin and other antimicrobial agents^24^.

### Phenotypic identification of OECs

For phenotypic identification of OECs in uninfected and infected cultures, the fixed cultures were incubated with rabbit polyclonal anti-p75 neurotrophin receptor antibodies (p75 NTR –1/400, Sigma) and an anti-smooth muscle α-Actin (anti-SM α-Actin – 1/100, Sigma). After reaction with the primary antibodies of interest, cells were incubated with goat anti-rabbit IgG and sheep or goat anti-mouse IgG secondary antibodies, labeled with either Alexa 488 or Cy3 (Life Technologies; Invitrogen, Carlsbad, CA, USA), washed in pH 7.4 PBS, mounted with N-propylgallate (Sigma) in PBS–glycerol and coverslipped.

### Assays of association and intracellular survival of S. pneumoniae in OECs or microglia

Association assays were performed in order to determine the total number of S. pneumoniae associated (both adhered and internalized bacteria) with OECs after 1, 3 and 5 h of interaction. This assay was conducted without antibiotics. For the intracellular survival assay, OECs or microglia were infected at a multiplicity of infection of 100:1 (bacteria:cell) with strain EF3030 in serum- and antibiotic-free DMEM/F-12. Cells were incubated for 3 h at 37 °C, and then free bacteria were removed by five washes with serum-free DMEM. The infected cultures were rinsed with 2% penicillin-streptomycin solution (Invitrogen, Carlsbad, CA, USA) for 1 h to kill S. pneumoniae cells adhered to the cellular surface. After this period, 3 washes were done and the cell cultures were lysed with 20 μM digitonin, serially diluting the contents of each well in Blanks buffer-PRAS, and plated onto sheep blood agar plates (Plast Labor, Rio de Janeiro, RJ, Brazil) for enumeration of colony-forming units (CFU).

### Interaction of *S. pneumoniae* with OECs or microglia cultures

When the cell cultures reached semi-confluence, interaction assays with *S. pneumoniae* were performed as previously described in detail^7^. After repeated washing to remove any residual FCS, the OEC cultures were infected for up to 5 h at 37 °C with suspensions of living *S. pneumoniae* cells in serum- and antibiotic-free DMEM/F-12. *S. pneumoniae* cells were detected by using a pneumococcal anti-serum (OMNI States Serum Institute, Copenhagen, Denmark) and/or stained with 0.1 mg/mL 4′,6-diamidinophenylindole (DAPI, Sigma) as already described^7^.

In order to investigate NO production, culture supernatants from uninfected and infected OEC or N13 cell cultures maintained for periods of 1, 3, and 5 h were analyzed for their nitrite content, using the Griess reaction. Highly purified bovine serum albumin (BSA) at 2% concentration was tested in place of fetal bovine serum (FBS) during the NO measurement and infection protocols. The supernatants, collected previously, were filtered through 0.22 μm pore membranes, aliquoted and stored at −80 °C. The concentration of nitrite, a stable metabolite of nitric oxide (NO), was measured in culture supernatants by the Griess assay as described earlier^25^. After 10 min incubation at room temperature, the optical density of a 1: 1 mixture of supernatant (100 μL) and Griess reagent (100 μL) was measured in a microtiter plate reader (Flow, Meckenheim, Germany) at 546 nm against a blank consisting of the medium without phenol red. A sodium-nitrite standard curve (0–100 mM) was generated in parallel.

For some samples, the infection was followed at 10, 20, 40, 60, and 180 min by fixation with 4% paraformaldehyde in PBS for 30 min at room temperature. After permeabilization of the cells with 0.3% PBS–Triton and blocking with 10% normal goat serum (NGS), the cultures were incubated with a rabbit polyclonal anti-nuclear factor-kappa B (NF-κB) p65 antibody (1:500; Santa Cruz Biotechnology, Santa Cruz, CA, USA), rabbit polyclonal anti-mannose receptor (1:100; Abcam, Cambridge, MA, USA) or with a rabbit anti-inducible nitric oxide synthase (iNOS) antibody (1:200; Cell Signaling Technology, Beverly, MA, USA). After the reaction with the primary antibodies of interest, cells were incubated with goat anti-rabbit IgG and sheep or goat anti-mouse IgG secondary antibodies, labeled with either Alexa 488 or Cy3, mounted with N-propylgallate in PBS-glycerol and coverslipped. To circumvent any potential bias that could induce iNOS expression in our culture system, such as contamination with outer-surface membrane lipopolysaccharides (LPS) that are present in almost all Gram-negative bacteria, we used polymyxin B (poly B; Sigma) as an inhibitor of LPS. Some assays were performed in the presence of 10 μg/mL of poly B. Since FSK increases the enhancement of LPS-induced iNOS expression in another type of glial cell^26^ and has also been added to the defined media as a culture requirement of neonatal rat OECs^2^, we evaluated whether this could be responsible for possibly stimulating the expression of iNOS.

In order to investigate the presumptive regulation of the pneumococcal infection in OECs, uninfected and infected cultures were incubated or not with rIFN-γ (100 U/mL) for 18 h and the percentage of infected cells was determined after the interaction step. The glass coverslips were then removed from each well and mounted on glass slides with N-propylgallate in PBS-glycerol. Samples were analyzed using a Zeiss epifluorescence photomicroscope (Zeiss, Jena, Germany) and a set of 200 cells was examined for the presence of *S. pneumoniae*. In addition, the percentage of cells with associated bacteria (adhered or internalized) was calculated as follows: number of infected cells/200 cells × 100.

### Quantitative RT-PCR analysis of iNOS mRNA expression in OEC cultures uninfected and infected by *Streptococcus pneumoniae*

Total RNA was extracted from infected and uninfected OEC cultures at a density of 3 × 10^6^ cells per dish using Trizol reagent (Invitrogen)^27^. RNA concentration was measured by spectrophotometry in a Nanodrop ND-1000 system. The synthesis of cDNA from mRNA was performed by using the SuperScrip III first-strand synthesis system RT-PCR kit (Invitrogen) according to the manufacturer’s instructions. The cDNA obtained served as a template for PCR with gene specific primer sets. PCR primers for target genes were purchased (Invitrogen). The following pairs of primers were used to determine mRNA iNOS levels: forward 5′- CAGCTGGGCTGTACAAACCTT-3′ and reverse 5′-CATTGGAAGTGAAGCGTTTCG-3′. GAPDH forward 5′-TGCACCACCACCTGCTTAGC-3′ and GAPDH reverse 5′-GGCATGGACTGTGGTC ATGAG-3′ were used for normalization. Relative mRNA levels were measured with a iTaq Universal SYBR green Supermix detection system using ABI 7500 Real-Time PCR (Applied Biosystems, Foster City, CA, USA). Quantitative RT-PCR was performed to measure the relative levels of expression of iNOS gene in the infected and uninfected OEC cultures. The entire set of experiments was performed three times. For each sample, the expression of each gene was normalized to the expression of the housekeeping gene GAPDH using the 2^−ΔΔCt^ method, where ΔCt = Ct, reference gene; Ct, target gene.

### Ultrastructural analysis of OECs and microglia after interaction with *S. pneumoniae*

Infected cultures were rinsed in PBS five times to remove nonadhered *S. pneumoniae* and immediately fixed in 2.5% (v/v) glutaraldehyde in 0.1 M sodium cacodylate buffer pH 7.2 at 4 °C for 1 h. Cells were scraped out of the dishes on ice, centrifuged at 900 × g for 10 min, and post-fixed in 1% osmium tetroxide (Sigma) in 0.1 M sodium cacodylate buffer containing 3.5% sucrose at 4 °C for 30 min. Cells were then washed in the same buffer, dehydrated in acetone, and embedded in PolyBed 812 (Polyscience, Philadelphia, PA, USA). Thin sections were obtained in an OmU3 Reichert ultramicrotome, stained with aqueous 2% uranyl acetate, and examined in a Zeiss EM 10C transmission electron microscope.

### Statistical analysis

Statistical analyses of the data from the assays of viability, NF-κB translocation and cell/bacteria association were performed with One-way ANOVA followed by the Tukey test for multiple comparisons. For measurement of nitrite concentration, Two-way ANOVA was applied, followed by Bonferroni post-test. Data from quantitative RT-PCR were compared by Student’s *t*-test of uninfected vs. infected OEC cultures. Data were analyzed using PRISM (Version 5.01, Graph Pad Software, La Jolla, CA, USA). *P* values equal to or less than 0.05 were considered statistically significant.

## Additional Information

**How to cite this article**: Macedo-Ramos, H. *et al*. *Streptococcus pneumoniae* resists intracellular killing by olfactory ensheathing cells but not by microglia. *Sci. Rep.* **6**, 36813; doi: 10.1038/srep36813 (2016).

**Publisher’s note:** Springer Nature remains neutral with regard to jurisdictional claims in published maps and institutional affiliations.
